# Reliability of intra-operative frozen section study in revision of infected hip arthroplasty

**DOI:** 10.1186/s42836-019-0016-2

**Published:** 2019-12-05

**Authors:** Karan Doshi, Deepesh Daultani, M. Ajith Kumar, Shantharam Shetty, Shailesh Pai

**Affiliations:** Tejasvini Hospital & SSIOT, Kadri Temple road, Kadri, Mangalore, Karnataka 575003 India

**Keywords:** Infection, Arthroplasty, Hip, Frozen sections

## Abstract

**Introduction:**

Frozen sections are extensively used to help in the diagnosis of periprosthetic joint infection during revision hip arthroplasty, though there are insufficient data in relation to its usefulness.

**Methods:**

Twenty-one patients with infected hip arthroplasties were operated in the form of one or two-staged revision hip arthroplasties. A frozen section was obtained intra-operatively and > 5 PMN’s/ HPF was considered as a positive indicator of infection. If the frozen section was reported negative (≤5 PMN’s/HPF), the revision prosthesis was implanted after a thorough debridement and a wash. If the frozen section was reported as positive, post the debridement; a non-articulating antibiotic-loaded cement spacer was implanted for 8 weeks, supplemented with 3 weeks of intravenous antibiotics and 3 weeks of oral antibiotics. This was followed by an antibiotic-free interval of 2 weeks. The patient was taken up for a revision surgery once the frozen section study was negative (≤5 PMN’s/HPF). The patients were followed up for a minimum of 1 year to a maximum of 2 years after the revision for any evidence of infection (assessed clinically, serologically, and radiologically).

**Results:**

Frozen section analysis of PMNs per high power field had a 100% specificity in our patients in detecting periprosthetic joint infection.

**Conclusion:**

Frozen section study is a safe, rapid, cheap and reliable intra-operative modality to diagnose periprosthetic joint infection.

## Introduction

Periprosthetic joint infection (PJI) of total hip arthroplasty is considered to be a formidable complication. Gallo et al. [1] estimated the periprosthetic joint infection rate ranged from 1 to 2% for a primary hip arthroplasty. The primary aim is to differentiate between PJI and aseptic loosening before taking the patient up for a revision surgery. This may be clinically difficult because of the low virulence and biofilm-forming ability of the pathogens. The history and clinical examination, although relevant, are questionable in terms of reliability. There are multiple new modalities to aid in the diagnosis, but presently there is no pre-operative diagnostic test that is 100% reliable for the diagnosis of PJI. Essential requirements before re-implantation include clinical, serological evidence of resolution of infection and a healthy intra-operative field. Unintended implantation of a revision prosthesis into an infected field is an unfortunate clinical, economic, social and medico-legal burden on the patient, surgeon and society due to misinterpretation of a PJI. On the contrary, classifying a non-infected hip arthroplasty as a PJI results in the patient enduring prolonged treatment with needless multiple surgeries.

Frozen section analysis is an affordable tool, and when done intra-operatively, it gives a rapid result and helps to differentiate between aseptic loosening and PJI. In this study we evaluated the reliability of intra-operative frozen section study in revision of infected hip arthroplasty.

## Materials and methods

All patients who were operated for revision of infected hip arthroplasty at our institution for a period of 2 years were included in the study after ethics committee approval. The patient was considered to have a periprosthetic joint infection (PJI) if at least one of the following criteria was present:

1. Raised erythrocyte sedimentation rate (ESR) & C-Reactive Protein (CRP) preoperatively as per the cut off values of 30 mm/hr and 10 mg/L respectively.
2. Presence of an active or quiescent sinus tract communicating with the joint.
3. Visible purulence of intraoperative periprosthetic tissue (as determined by the surgeon).

The patients were informed about the nature of the study and consent for the procedure was taken from the patients.

## Operative protocol (Fig. 1)

All patients with periprosthetic infection were treated with prosthesis removal and thorough debridement. Tissue samples were harvested by sharp dissection from the joint pseudocapsule, membrane of a loose component, peculiar pigmented tissue, or any areas of bony erosion. All tissue samples for frozen section were obtained from multiple surgical sites including a minimum of 3 samples each from acetabulum and femoral side respectively. Five sections were taken from each femoral and acetabular sample. The sections were stained with hematoxylin and eosin. Microscopic examination of the slides was done to check for polymorphonuclear leukocytes (PMNs). The mounted slides were first examined under low power to choose the five areas with most PMNs. These five areas were rechecked under high power (× 40) and the PMN count of the most cellular area was recorded. A frozen section was considered positive by the presence of more than 5 PMNs per high-power field (HPF) in at least 5 separate HPFs, with surface inflammatory exudate and fibrin excluded based on Mirra’s criteria [2] (adapted by Feldman [3]).

**Fig. 1:**
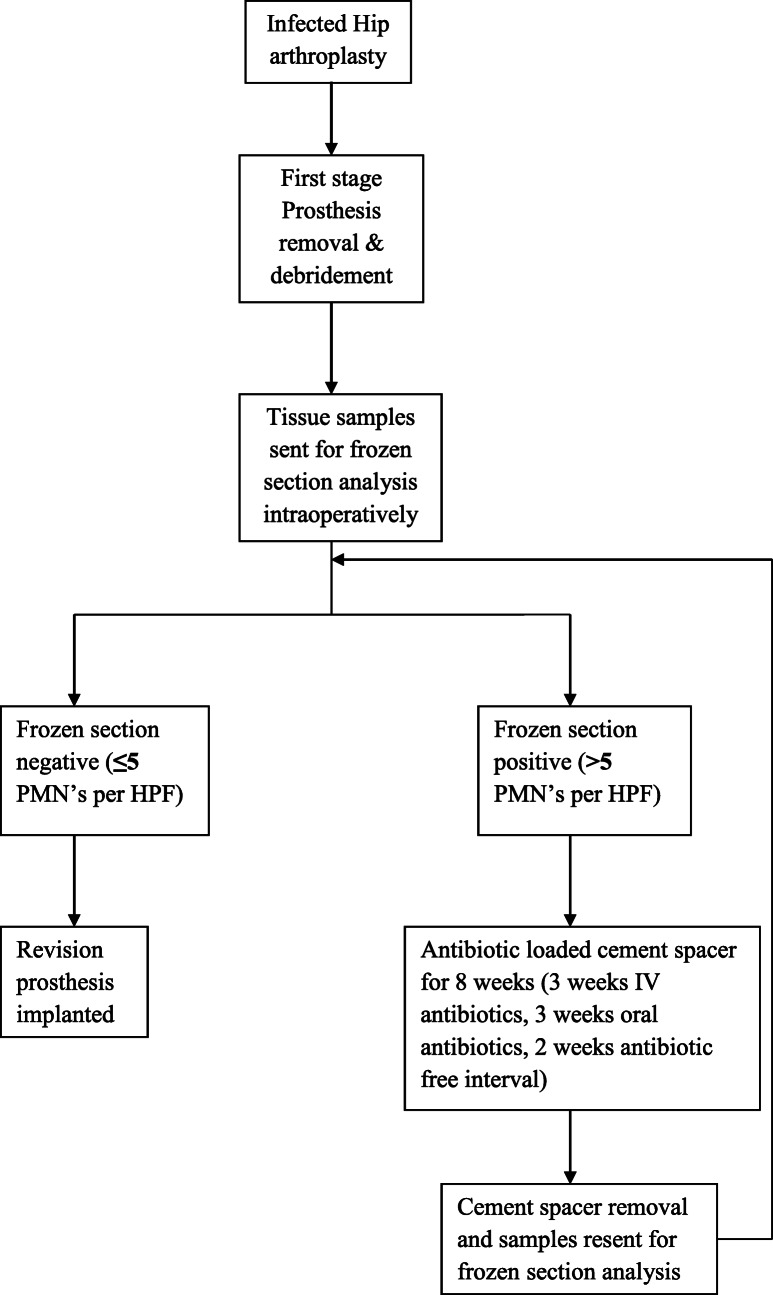
Operative protocol

If the frozen section was reported negative (≤5 PMN’s per HPF), the revision prosthesis was implanted after a thorough debridement and a wash.

**Fig. 2:**
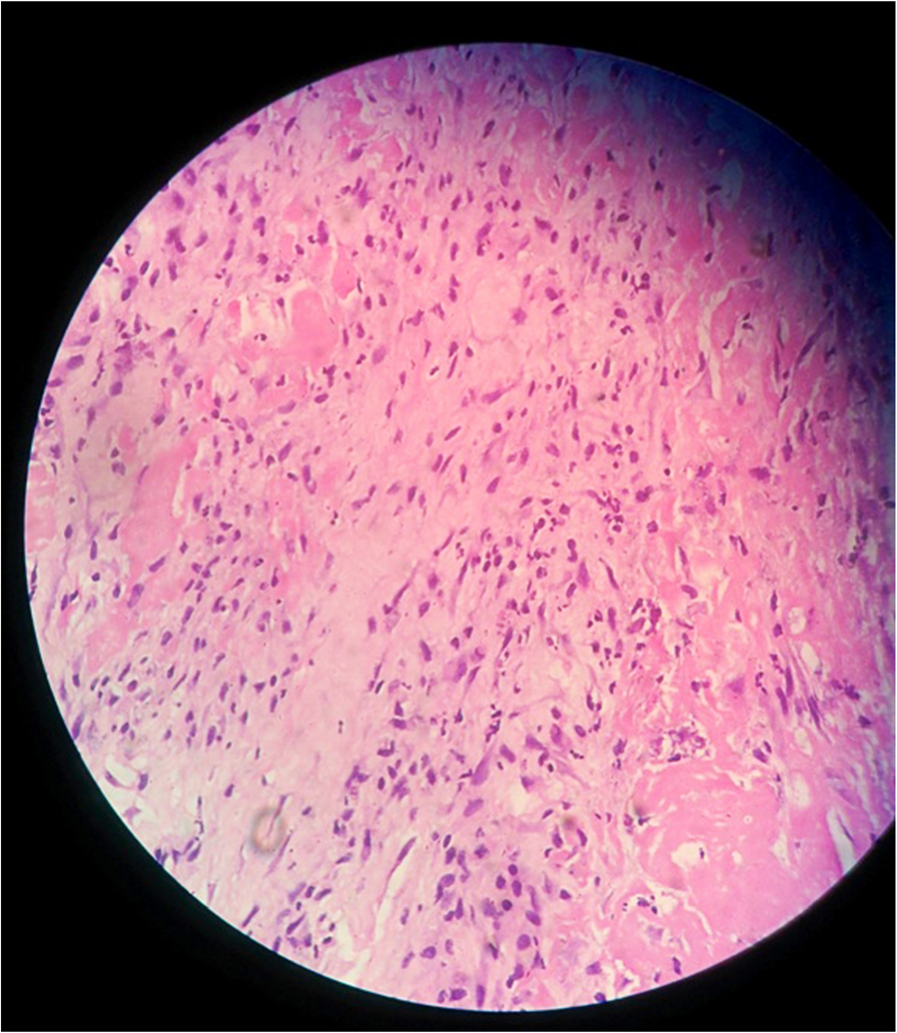
12–14 PMNs/ HPF on frozen section of femoral sample from a patient

If the frozen section was reported to be positive (Fig. 2), post the debridement, an antibiotic-loaded cement spacer was prepared on a rush pin. 3–4.5 g of Vancomycin was added to 40 g of Polymethyl-metha acrylate (PMMA) and hand- moulded onto the rush pin. This static non-articulating cement spacer was implanted for 8 weeks supplemented with 3 weeks of intravenous antibiotics and 3 weeks of oral antibiotics. This was followed by an antibiotic-free interval of 2 weeks. At the time of the second stage surgery, the serological tests were repeated. The patient was considered to have persistent infection if the serological markers (ESR, CRP) were raised and the patient was taken up for cement spacer removal and redebridement. The tissue samples were re-sent for frozen section analysis intraoperatively. If the frozen section was negative (≤5 PMN’s per HPF), re-implantation was performed with the revision hip prosthesis after a thorough debridement and wash. The patients were followed up for a minimum of 1 year to a maximum of 2 years after the revision for any evidence of infection (assessed clinically serologically and radiologically).

If the frozen section was positive again at the time of the second-stage surgery, the patient underwent another stage of antibiotic-loaded cement spacer application and 6 weeks of antibiotics according to the protocol listed above. The patient was taken up for the third-stage surgery after 2 weeks of antibiotic-free interval wherein the frozen section was repeated and the revision hip replacement was done.

## Results & observations

Twenty-three patients were operated for revision of infected hip arthroplasty. Out of those, 2 patients were lost to follow up, thus 21 patients were included in the study. The subjects included 5 females and 16 males, with a mean age of 55.57 years (range 41–68 years). The mean presentation since index surgery was 5.9 years (range 2–15 years). Out of the 21 patients with infected hip arthroplasty, 5 patients had an Austin Moore prosthesis, 7 patients had a bipolar prosthesis, and 9 patients had a total hip prosthesis. Eleven out of the 21 patients had a cemented prosthesis. Four out of the 21 patients had a sinus tract, all of which were not actively discharging. Intra-operative purulence was present in 7 out of 21 patients in the first stage of revision arthroplasty. The ESR and CRP were high (> 30 mm/hr and > 10 mg/L respectively) in all 21 patients in the first stage. Four patients out of 15 had high ESR in the second stage. Four patients out of 15 had high CRP in the second stage. Six patients had negative frozen section (≤5PMN’s/HPF) values in the first and thus primary one stage revision hip arthroplasty was undertaken. Fifteen patients had a positive frozen section (>5PMN’s/HPF) in the first stage and were treated with prosthesis removal and cement spacer insertion for 8 weeks. In the second stage, out of 15 patients, 14 underwent revision arthroplasty, while 1 patient underwent reapplication of the cement spacer. As per the follow up ESR & CRP values, clinically and radiologically no patients had any evidence of infection. The average follow-up was 17.04 months (range 12–24 months). One patient had persistently raised ESR (34 mm/hr) which may be attributable to other causes. On analysis, since no patient had any evidence of infection on follow-up, frozen section analysis of PMN’s per high power field had a 100% specificity in our patients in detecting periprosthetic joint infection.

## Discussion

Periprosthetic joint infection is the most disastrous complication of a total hip arthroplasty. The infection endangers the functional utility of the hip joint, although rarely. It also threatens the life of the patient. The primary step in managing a PJI is to diagnose it. Diagnosing a periprosthetic joint infection is a challenge preoperatively. Equivocal results have been found upon comparison of multiple modalities of diagnosis.

Although most failures tend to occur within the first year after implantation, infections could take place after many years of apparently successful treatment [4]. In our study, the mean presentation of periprosthetic hip infection was 5.90 years after the primary surgery (range 2–15 years). None of the patients had undergone a revision hip arthroplasty for the same hip before or undergone any surgery in the last 1 year which could have contributed to the increased levels of serological markers at the time of presentation. Four patients presented with a quiescent non-draining sinus. This is considered to be a major diagnostic criterion of PJI as per the Musculoskeletal Infection Society criteria [5]. Seven patients had intraoperative purulence and all those went on for a two-stage revision arthroplasty. The so-called standard for diagnosing infection at the site of a total hip arthroplasty has long been intra-operative cultures [6, 7]. An obvious difficulty with intra-operative cultures is the time required to obtain useful results. This delay prevents intra-operative cultures from being useful in decision-making during an equivocal procedure.

The technique of using frozen section histology as an intra-operative tool was first mentioned by Charosky et al. [8], who concluded that if at the time of re-operation, frozen section tissues from the pseudocapsule showed acute inflammatory changes or severe chronic inflammation, that could be presumptive evidence of infection. Most of the present literature compares frozen section histology to intraoperative culture as a gold standard. Considering the fallacies of intraoperative culture, we tested frozen section histology as a separate entity and calculated the positive and negative outcomes for the same. With this we would know if the test is reliable to rule out presence of infection at the time of re-implantation with the prosthesis. Frozen section analysis is unfortunately not foolproof and has its own drawbacks. The surgeon, who is collecting the sample from the suspicious areas, is doing so with naked eyes. Thus, the tissue sample selection is subjective. The sample has to be handled with care while being transferred to the pathology department. A skilled and experienced pathologist should analyse all the tissue sections. The analysis may skew in any direction if any of the above-mentioned subjective parameters are not managed carefully.

A negative result on both ESR & CRP is extremely good in ruling out active periprosthetic joint infection. A positive result on both tests; is more reliably indicative of periprosthetic joint infection compared to a positive result on just one test. In a study of 414 revision total hip arthroplasties and 538 total knee arthroplasties, Mc Arthur et al. [9] reported that the incidence of seronegative PJI was 4%, and the sensitivity of ESR and CRP was 81 and 93%, respectively. They claimed that a subset of patients with PJI will present with a normal ESR and CRP. Once diagnosed, most seronegative PJIs were successfully treated with a two-stage revision.

Frozen section study was done at both stages of revision at our institute. Fifteen patients who had positive frozen section (> 5 PMN’s / HPF) in the first stage were treated by prosthesis removal and cement spacer application. Six patients had negative frozen section (≤5PMN’s/HPF) values in the first stage and thus primary one stage revision hip arthroplasty was undertaken. Fourteen out of the 15 patients in the second stage had a negative frozen section result and underwent revision hip arthroplasty. One patient underwent debridement and repeated cement spacer application since the frozen section was positive even on the second stage. The patient eventually underwent revision hip arthroplasty in the subsequent stage. One serial follow-up, for a minimum of 1 year and a maximum up to 2 yearsshowed that no patient had any clinical, serologically and radiological evidence of infection. Post analysis, the frozen section analysis predicted all true negatives and showed a 100% specificity in diagnosing PJI. One patient had elevated ESR (34 mm/hr) duringa 1-year follow-up. The CRP and other parameters were all within normal range. There were no other signs of infection, thus this raised ESR could be attributable to other causes.

Four patients in the second stage of revision arthroplasty had elevated serological markers. Two patients had both increased ESR and CRP while the other two patients had only ESR or CRP respectively. Intra-operatively, the frozen section analysis of these 4 patients was negative and a revision prosthesis was implanted after cement spacer removal even though the serological values were raised. On follow-up, these patients had no serological, clinical or radiological signs of infection.

In a systematic review and a meta-analysis of longitudinal studies, Tsaras et al. compared frozen section histologic results with simultaneously obtained microbiologic culture[10], they concluded that intra-operative frozen sections of periprosthetic tissues performed well in predicting a diagnosis of culturally-positive periprosthetic joint infection but had moderate accuracy in ruling out the diagnosis.

In our series, 3 patients were managed at another center with oral antibiotics for their apparent infection, one had stopped the intake of antibiotics 1 week prior to presentation to our centre; while the other 2 patients still continued to take the oral antibiotics at the time of presentation. These patients had relatively lower ESR and CRP values compared to the rest of the patients in the study, but their frozen section values ranged from 8 to 12 PMN’s / HPF at the time of the first- stage revision arthroplasty. All these 3 patients were managed with a two-stage revision arthroplasty.

Della Valle et al. [11] reported that intra-operative analysis of frozen sections at the time of re-implantation had a sensitivity of 25%, a specificity of 98%, a positive predictive value of 50%, a negative predictive value of 95% and an accuracy rate of 94%. Sensitivity was increased to 75% when the authors changed the criteria for a positive result to at least one polymorphonuclear leukocytes found in the frozen section. However, both specificity and accuracy decreased to 80%. We used Mirra’s [2] criteria and classified a frozen section result as positive if > 5 PMN’s/ HPF were present. In a meta-analysis published in 2013 [12], it was found that both thresholds, five and ten polymorphonuclear leukocytes per high-power field, yielded acceptable results in frozen section tests for periprosthetic infection, although a threshold of ten had a greater specificity, without decreasing sensitivity. The meta-analysis indicated that although both the two thresholds are stable and effective, a threshold of ten polymorphonuclear leukocytes per high-power field is better for diagnosing periprosthetic infections.

## Limitations of the study

- Small sample size.
- Short duration of follow-up.
- No comparison to culture.
- No intra- and inter-observer variability for frozen section analysis.

## Conclusion

Intra-operative frozen section study is a quick & reliable indicator in predicting a diagnosis of PJI with good accuracy and in ruling out this diagnosis. Frozen section study should thus be considered a relevant part of the challenging diagnostic work-up for patients undergoing revision hip arthroplasty, although intra- and inter-observer variability needs to be considered.

## Data Availability

Please contact author for data requests.

## Abbreviations

CRP: C-Reactive Protein
ESR: Erythrocyte sedimentation rate
HPF: High-power field
PJI: Periprosthetic joint infection
PMMA: Polymethyl-metha acrylate
PMN: Polymorphonuclear leukocytes
