# Correction to: Reliability of intra-operative frozen section study in revision of infected hip arthroplasty

**DOI:** 10.1186/s42836-019-0019-z

**Published:** 2019-12-18

**Authors:** Karan Doshi, Deepesh Daultani, M. Ajith Kumar, Shantharam Shetty, Shailesh Pai

**Affiliations:** Tejasvini Hospital & SSIOT, Kadri Temple road, Kadri, Mangalore, Karnataka 575003 India

**Correction to: Arthroplasty (2019) 1:15**

**https://doi.org/10.1186/s42836-019-0016-2**

In the original publication of this article [1], Fig. 1 is wrong due to a typesetting mistake. The correct figure 1 should be as follows.

**Fig. 1:**
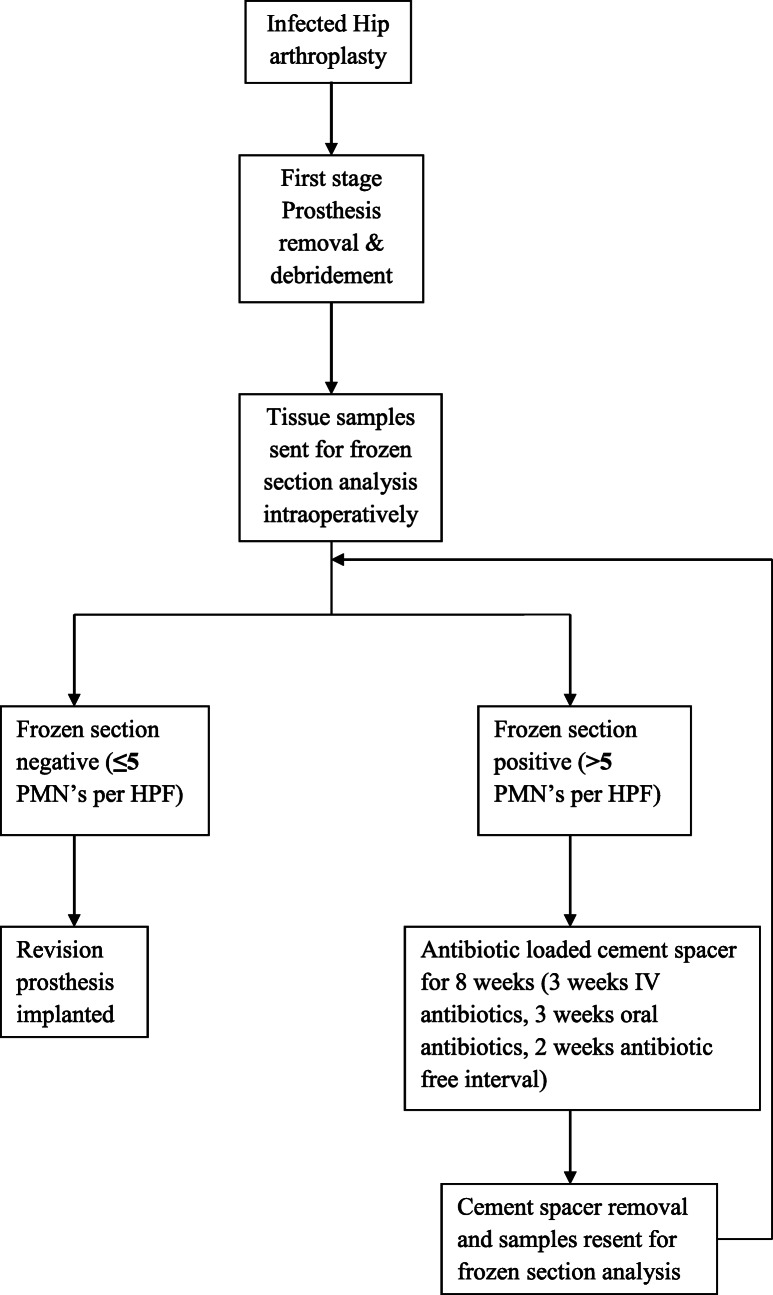
Operative protocol

The original article has been corrected.

## References

[1] Doshi K (2019). Reliability of intra-operative frozen section study in revision of infected hip arthroplasty. Arthroplasty.

